# Myosteatosis and aortic calcium score on abdominal CT as prognostic markers in non-dialysis chronic kidney disease patients

**DOI:** 10.1038/s41598-024-58293-3

**Published:** 2024-04-02

**Authors:** Ahyun Kim, Chul-min Lee, Bo-Kyeong Kang, Mimi Kim, Jong Wook Choi

**Affiliations:** 1 Department of Radiology, Hanyang University Medical Center, 222-1 Wangsimni-ro, Seongdong-gu, Seoul, 04763 Republic of Korea; 2 Department of Internal Medicine, Hanyang University Medical Center, 222-1 Wangsimni-ro, Seongdong-gu, Seoul, Republic of Korea

**Keywords:** Kidney diseases, Biomarkers

## Abstract

We aimed to examine the relationship between abdominal computed tomography (CT)-based body composition data and both renal function decline and all-cause mortality in patients with non-dialysis chronic kidney disease (CKD). This retrospective study comprised non-dialysis CKD patients who underwent consecutive unenhanced abdominal CT between January 2010 and December 2011. CT-based body composition was measured using semiautomated method that included visceral fat, subcutaneous fat, skeletal muscle area and density, and abdominal aortic calcium score (AAS). Sarcopenia and myosteatosis were defined by decreased skeletal muscle index (SMI) and decreased skeletal muscle density, respectively, each with specific cutoffs. Risk factors for CKD progression and survival were identified using logistic regression and Cox proportional hazard regression models. Survival between groups based on myosteatosis and AAS was compared using the Kaplan–Meier curve. 149 patients (median age: 70 years) were included; 79 (53.0%) patients had sarcopenia and 112 (75.2%) had myosteatosis. The median AAS was 560.9 (interquartile range: 55.7–1478.3)/m^2^. The prognostic factors for CKD progression were myosteatosis [odds ratio (OR) = 4.31, *p* = 0.013] and high AAS (OR = 1.03, *p* = 0.001). Skeletal muscle density [hazard ratio (HR) = 0.93, *p* = 0.004] or myosteatosis (HR = 4.87, *p* = 0.032) and high AAS (HR = 1.02, *p* = 0.001) were independent factors for poor survival outcomes. The presence of myosteatosis and the high burden of aortic calcium were significant factors for CKD progression and survival in patients with non-dialysis CKD.

## Introduction

Chronic kidney disease (CKD) has been recognized as a significant global health concern with increasing prevalence. Approximately 700 million people worldwide are affected by some form of CKD, and 1.2 million deaths from CKD were recorded in 2017^1,2^. Theoretically, CKD is a progressive condition without a cure, eventually leading to end-stage renal disease (ESRD) or death. Therefore, CKD treatment is geared toward slowing the progression of the disease or modifying risk factors. The early identification of risk factors for CKD progression may contribute to preventing complications from CKD and the progression to ESRD or death.

Abdominal computed tomography (CT) scans are commonly used for various medical indications, including CKD. These scans provide not only detailed images of the abdomen but also valuable information on body composition. The opportunistic use of body composition data from abdominal CT scans has become an area of increasing interest as it allows for valuable data that would otherwise require additional imaging or testing^3–5^.

The body composition data obtained from abdominal CT scans already have shown significant promise as prognostic tools for the risk of adverse clinical outcomes, such as future adverse cardiovascular events and death, in patients with CKD^6–12^. However, the studies focused primarily on patients with ESRD who were undergoing renal replacement therapy, a group known for high mortality and morbidity^7–10,12^. Nevertheless, alterations in body composition, such as changes in visceral fat, muscle mass, and vascular calcification, are known to occur even in earlier stages of the disease^13–15^. As such, further investigations of the prognostic value of these body composition data in patients with non-dialysis CKD are needed. In addition, the relationship between body composition data and changes in renal function in patients with CKD also requires attention. While some studies have investigated the correlation between body composition data and renal function in patients with CKD, these were limited. A few studies evaluated only a single time point of renal function in patients with CKD without analyzing longitudinal changes^16,17^. They also used only a single type of body composition data (e.g., visceral fat area) instead of analyzing comprehensive data^18,19^.

In this study, we analyzed the body composition data from abdominal CT scans, including visceral fat area (VFA), subcutaneous fat area (SFA), skeletal muscle area, skeletal muscle density, and abdominal aortic calcium score (AAS), in the semiautomated method. We investigated the relationship between these body composition data and longitudinal changes in renal function, as well as the relationship with all-cause mortality in patients with non-dialysis CKD.

## Materials and methods

### Study population

**Figure 1:**
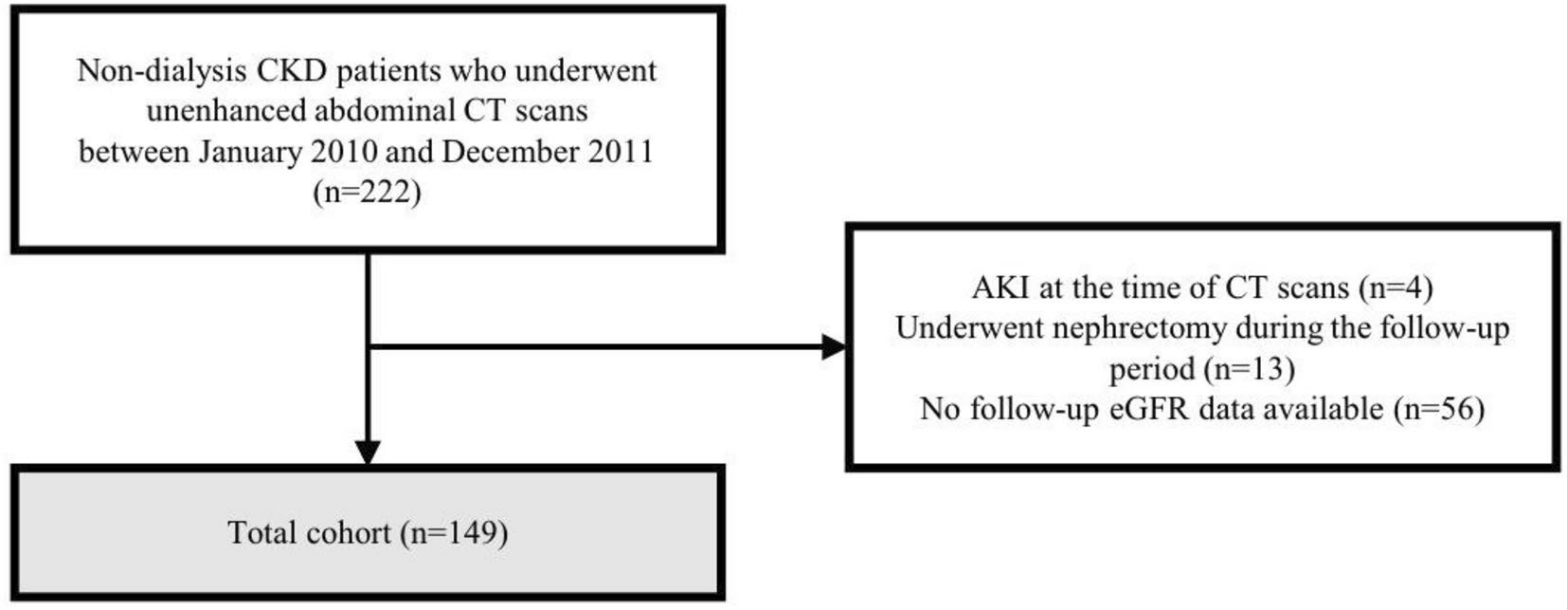
The flow chart of patient inclusion.

The Institutional Review Board of Hanyang University Hospital approved this retrospective study and the requirement for informed consent was waived due to its retrospective nature (IRB No. HYUH 2023-05-031). All methods followed the relevant guidelines and regulations. The study cohort comprised 222 patients who underwent consecutive unenhanced abdominal CT scans between January 2010 and December 2011 and were clinically diagnosed with non-dialysis CKD. Patients with acute kidney injury at the time of the CT scans (n = 4) and those who underwent nephrectomy during the follow-up period (n = 13) were excluded. Patients who did not have available follow-up data on estimated glomerular filtration rate (eGFR) (n = 56) were also excluded. The flow chart of patient inclusion is shown in Fig. 1.

For each patient, the baseline characteristics and biochemical test data at the time of the CT scans were collected. Indications for CT scan, data on age, sex, height, weight, body mass index, and the presence of hypertension and diabetes mellitus were collected from electronic medical records. Further, data on biochemical parameters, serum sodium, potassium, chloride, total CO_2_, creatinine, blood urea nitrogen (BUN) levels, and eGFR, at the time of the CT scans were collected. eGFR was calculated from serum creatinine using the CKD Epidemiology Collaboration (CKD-EPI) equation^20^.

### Image acquisition

Multidetector row CT scans were performed with one of two multidetector CT machines (Sensation 16, Siemens Healthineers; Brilliance 64, Philips Healthcare) without contrast enhancement. The scanning parameters were as follows: 120 kV (peak), 189–200 mAs, 5-mm slice thickness, and table speed of 26.5–39.37 mm/rotation (pitch = 0.828–1.07). Transverse images were reconstructed using a slice thickness and reconstruction interval of 5 mm. All of images were evaluated using a picture archiving and communication system (PACS).

### Image analysis

#### VFA, SFA, skeletal muscle area, and skeletal muscle density

**Figure 2:**
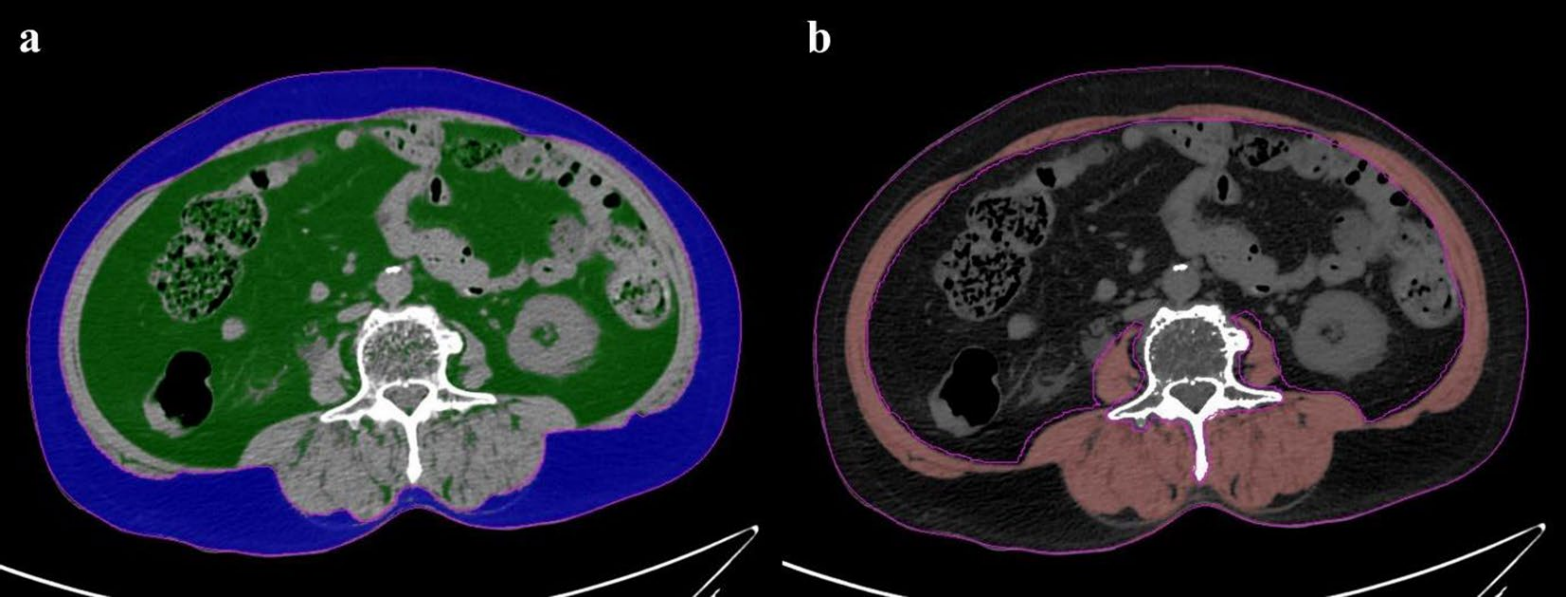
Example of the semiautomated segmentation of the subcutaneous fat area, visceral fat area, and skeletal muscle area at the L3 vertebral level. (**a**) The subcutaneous fat is marked blue and the visceral fat, green. (**b**) The skeletal muscle area is marked red. To determine skeletal muscle density, the mean Hounsfield unit (HU) value of the skeletal muscle area was automatically calculated.

Measurements of body composition data were performed by an experienced abdominal radiologist (13 years of experience of abdominal imaging). To quantify the VFA, SFA, and skeletal muscle area, axial CT images at the level of the third lumbar vertebra (L3) were analyzed via a semiautomated method using the Aquarius iNtuition viewer (version 4.4.13, TeraRecon). With this software, the VFA, SFA, and skeletal muscle area can be quantified automatically using the predetermined thresholds of Hounsfield unit (HU) values. Values from -29 to 150 HU were applied for skeletal muscle, while values from -190 to -30 HU were used for fat (Fig. 2). The automatic outlines were hand-adjusted by the analyzer. For normalization of the skeletal muscle area, the skeletal muscle index (SMI) was calculated according to the following formula: skeletal muscle area (cm^2^)/height^2^ (m^2^). Skeletal muscle density was also automatically measured as the mean HU value of skeletal muscle area at the L3 level. Sarcopenia was defined as SMI ≤ 52.4 cm^2^/m^2^ for men and ≤ 38.5 cm^2^/m^2^ for women based on a study by Prado et al.^21^. Myosteatosis was determined based on the skeletal muscle density of < 41 HU for body mass index (BMI) < 25 kg/m^2^ and < 33 HU for BMI ≥ 25 kg/m^2^ according to a study by Martin et al.^22^.

#### Abdominal aortic calcium score (AAS)

The present study also utilized the Aquarius iNtuition viewer software for AAS. Abdominal CT slices caudal to the crus of the diaphragm and cranial to the aortic bifurcation were manually selected. The definition of aortic calcium in this study involved an area comprising a minimum of three consecutive pixels with a CT density > 30 HU. The degree of abdominal aortic calcium was defined via the Agatston score method. To calculate the Agatston score, each calcified lesion area (in mm^2^) was multiplied by a density factor ranging from 1 to 4, depending on the maximal HU of the lesion as described by Agatston^23^. The sum of these products for all calcified lesions was the total AAS. Normalized AAS was calculated according to the following formula to calibrate the height: AAS/height^2^ (m^2^).

### Outcomes

The primary outcome of the study was CKD progression. To estimate the decline in eGFR for each patient, we collected the last recorded eGFR values from their last visits or prior to starting renal replacement therapy, with a minimum interval of 5 months since the first eGFR measurement. The annual decline in eGFR for each patient was estimated by dividing the change in eGFR by the duration of their follow-up period (year). We defined progression of CKD as an eGFR decline of > 5 mL/min/1.73 m^2^/year in line with the Kidney Disease Improving Global Outcomes definition^24^. As a secondary outcome, we assessed the all-cause mortality of patients by reviewing their medical records.

### Statistical analysis

All statistical analyses were performed with commercially available software (SPSS Statistics version 27.0, IBM Corp.; and MedCalc Software, version 19.0.4). To evaluate data distribution, the Shapiro–Wilk test was used. Normally distributed continuous data are presented as mean with standard deviation, and non-normally distributed data are presented as median with interquartile range (IQR). Categorical data are presented as frequencies and percentages. To evaluate group differences between males and females, we chose appropriate statistical tests among the independent Student’s t-test, Mann–Whitney U test, or chi-square test. Univariate and multivariate logistic regression models were used to evaluate the influence of body composition data on the progression of CKD. The linearity between the continuous independent variables and the logit of the dependent variable was checked using the Box-Tidwell test. The non-linear independent variable was log-transformed to achieve linearity. Univariate and multivariate Cox proportional hazard regression models were used to identify variables that influenced overall survival (OS). The Kaplan–Meier curve was used to compare OS between groups classified based on the AAS and skeletal muscle density. Optimal cut-off values of the AAS and skeletal muscle density were calculated via receiver operating characteristic analysis and maximized Youden’s index. Between-group statistical significance was calculated using the log-rank test. Statistical significance was defined as *p* < 0.05.

### Ethics approval

The Institutional Review Board of Hanyang University Hospital approved this study.

## Results

### Patient characteristics

The final study cohort included 149 patients (82 males and 67 females) with a median age of 70 (IQR, 59–76) years. Their clinical characteristics are presented in Table 1. Of the patients, 121 (81.2%) had hypertension and 85 (57.0%) had diabetes mellitus. The median baseline eGFR was 58 (IQR, 43–71) mL/min/1.73 m^2^. A significant proportion of patients had sarcopenia and myosteatosis, with 79 patients (53.0%) and 112 patients (75.2%), respectively. The median value of AAS was 560.9 (IQR, 55.7–1478.3) /m^2^.

**Table 1:** Patient characteristics.

Clinical characteristics	Total
Number of patients	149
Age, years	70 (59–76)
BMI, kg/m^2^	24.2 ± 3.9
Hypertension (%)	121 (81.2)
Diabetes mellitus (%)	85 (57.0)
Body composition data	
VFA, cm^2^	160.1 ± 70.0
SFA, cm^2^	118.0 (84.1–153.5)
Visceral-to-subcutaneous fat ratio	1.4 (0.9–2.0)
Skeletal muscle area, cm^2^	117.0 (95.7–143.5)
Skeletal muscle density, HU	30.4 ± 8.1
SMI, cm^2^/m^2^	45.2 (38.8–51.8)
Sarcopenia (%)	79 (53.0)
Myosteatosis (%)	112 (75.2)
AAS, 1/m^2^	560.9 (55.7–1478.3)
Laboratory findings	
Sodium, mEq/L	139 (137–141)
Potassium, mEq/L	4.1 (3.8–4.5)
Chloride, mEq/L	105 (102–107)
Total CO_2_, mEq/L	23.2 (20.9–26.4)
Creatinine, mg/dL	1.2 (0.9–1.6)
eGFR, mL/min/1.73 m^2^	58 (43–71)
BUN, mg/dL	21.0 (16.0–29.0)
Albumin, g/dL	3.7 ± 0.6
CRP, mg/dL	0 (0–2.9)
Endpoint	
Median annual eGFR decline, mL/min/1.73 m^2^	2.7 (0.7–5.4)
Median annual eGFR decline ≥ 5 mL/min/1.73 m^2^ (%)	43 (28.9)
Death (%)	28 (18.8)

Data are presented as mean ± standard deviation, median (interquartile range), or number of participants (percentage).

*BMI* body mass index, *VFA* visceral fat area, *SFA* subcutaneous fat area, *SMI* skeletal muscle index, *AAS* abdominal aortic calcium score, *eGFR* estimated glomerular filtration rate, *BUN* blood urea nitrogen, *CRP* C-reactive protein.

The reasons for the included abdominal CT scans are as follows: abdominal pain (n = 48), fever/infection focus evaluation (n = 24), evaluation for liver function test abnormality or work-up for known chronic liver disease (n = 15), follow-up for benign disease (n = 10), hematuria (n = 7), hematochezia (n = 4), trauma (n = 3), post-procedural complication work-up for renal biopsy (n = 2), and others (n = 36).

We followed up patients for a median period of 90 (range, 5–151) months to track changes in their eGFR values. For survival analysis, the median follow-up period was 100 (range, 14–151) months. The median value of annual eGFR decline was 2.7 (IQR, 0.7–5.4) mL/min/1.73 m^2^. During the follow-up period, progression of CKD, defined as annual eGFR decline of > 5 mL/min/1.73 m^2^, was observed in 43 (28.9%) patients. All-cause mortality occurred in 28 (18.8%) patients. The 1- and 5-year mortality rates were 0% (0 of 149) and 4.7% (7 of 149), respectively.

### Sex differences in body composition data

Table 2 provides a statistical summary of body composition data for males and females. Except for the AAS, there were significant differences in the majority of body composition variables between the two groups. Males had significantly greater VFA (171.8 cm^2^ vs. 145.8 cm^2^, *p* = 0.024) and visceral-to-subcutaneous fat ratio values (1.7 vs. 1.0, *p* < 0.001), while females had significantly higher SFA values (149.0 cm^2^ vs. 96.9 cm^2^, *p* < 0.001). Males had greater skeletal muscle area (136.5 cm^2^ vs. 94.7 cm^2^, *p* < 0.001) and SMI (50.3 cm^2^/m^2^ vs. 40.1 cm^2^/m^2^, *p* < 0.001), but males also had a higher prevalence of sarcopenia (53% vs. 26%, *p* = 0.002), as defined by Prado et al.^21^. In contrast, females demonstrated significantly lower skeletal muscle density (25.7 HU vs. 34.2 HU, *p* < 0.001) and a substantially higher prevalence of myosteatosis (94% vs. 59.8%, *p* < 0.001).

**Table 2:** Sex difference of body composition data.

	Male	Female	*p* value
Number of patients	82	67	
Body composition data			
VFA, cm^2^	171.8 ± 74.5	145.8 ± 61.7	0.024
SFA, cm^2^	96.9 (67.8–123.5)	149.0 (112.0–189.0)	< 0.001
Visceral-to-subcutaneous fat ratio	1.7 (1.4–2.4)	1.0 (0.7–1.2)	< 0.001
Skeletal muscle area, cm^2^	136.5 (119.0–154.3)	94.7 (84.8–109.0)	< 0.001
Skeletal muscle density, HU	34.2 ± 7.2	25.7 ± 6.8	< 0.001
SMI, cm^2^/m^2^	50.3 (42.8–55.5)	40.1 (36.5–44.6)	< 0.001
Sarcopenia (%)	53 (64.6)	26 (38.8)	0.002
Myosteatosis (%)	49 (59.8)	63 (94.0)	< 0.001
AAS, 1/m^2^	531.2 (97.5–1749.8)	592.6 (24.3–1247.4)	0.577

Data are presented as mean ± standard deviation, median (interquartile range), or number of participants (percentage).

*VFA* visceral fat area, *SFA* subcutaneous fat area, *SMI* skeletal muscle index, *AAS* abdominal aortic calcium score.

### Prognostic factors for decrease of renal function

Table 3 provides results of the univariate and multivariate logistic regression analyses to evaluate the prognostic factors for CKD progression. In the univariate analysis, the presence of myosteatosis [odds ratio (OR) = 4.41, 95% confidence interval (CI): 1.46–13.35, *p* = 0.009], high AAS (OR = 1.02, 95% CI: 1.00–1.04, *p* = 0.028), and high baseline eGFR value (OR = 1.02, 95% CI: 1.01–1.04, *p* = 0.002) were significant prognostic factors predicting CKD progression. These three parameters remained significant in the multivariate logistic analysis (OR = 4.31, 95% CI: 1.36–13.69, *p* = 0.013 for myosteatosis; OR = 1.03, 95% CI: 1.01–1.06, *p* = 0.019 for AAS; and OR = 1.03, 95% CI: 1.01–1.05,* p* = 0.001 for eGFR).

**Table 3:** Univariate and multivariate logistic regression analysis for chronic kidney disease progression.

Variable	Univariate analysis			Multivariate analysis		
	OR	95% CI	*p* value	Adjusted OR	95% CI	*p* value
Sex (male)	0.96	0.47, 1.95	0.903			
Age, year	1.01	0.98, 1.03	0.627			
BMI, log(kg/m^2^)	0.28	0.00, 41.39	0.620			
HTN (absence)	0.46	0.20, 1.08	0.074			
DM (absence)	1.22	0.59, 2.51	0.592			
VFA, cm^2^	1.00	0.99, 1.00	0.668			
SFA, cm^2^	1.00	0.99, 1.01	0.640			
SMI, cm^2^/m^2^	0.98	0.95, 1.02	0.387			
Visceral-to-subcutaneous fat ratio	0.91	0.67, 1.23	0.535			
Skeletal muscle area, cm^2^	1.00	0.98, 1.01	0.368			
Skeletal muscle density, HU	0.97	0.92, 1.01	0.122			
Sarcopenia (absence)	0.79	0.39, 1.60	0.515			
Myosteatosis (absence)	4.41	1.46, 13.35	0.009	4.31	1.36, 13.69	0.013
AAS (100/m^2^ increment)	1.02	1.00, 1.04	0.028	1.03	1.01, 1.06	0.019
Creatinine, mg/dL	0.65	0.37, 1.14	0.134			
eGFR, mL/min/1.73 m^2^	1.02	1.01, 1.04	0.002	1.03	1.01, 1.05	0.001
BUN, mg/dL	0.98	0.96, 1.01	0.217			
Albumin, g/dL	0.81	0.42, 1.57	0.537			
CRP elevation (absence)	0.58	0.28, 1.20	0.143			

The reference category for each categorical variable is in the round brackets in the first column.

*BMI* body mass index, *VFA* visceral fat area, *SFA* subcutaneous fat area, *SMI* skeletal muscle index, *AAS* abdominal aortic calcium score, *eGFR* estimated glomerular filtration rate, *BUN* blood urea nitrogen, *CRP* C-reactive protein.

### Prognostic factors for survival

The outcomes of the univariate and multivariate Cox proportional hazard regression models for OS are presented in Table 4. In the univariate analysis, mortality was predicted by older age [hazard ratio (HR) = 1.04, 95% CI: 1.00–1.07, *p* = 0.048] and elevated BUN (HR = 1.01, 95% CI: 1.00–1.02, *p* = 0.001). Among the body composition data, high AAS (HR = 1.02, 95% CI: 1.01–1.03, *p* < 0.001), low skeletal muscle density (HR = 0.94, 95% CI: 0.89–0.98, *p* = 0.009), and presence of myosteatosis (HR = 5.66, 95% CI: 1.34–23.88, *p* = 0.018) were significant prognostic factors for mortality as well. To avoid collinearity, two related variables, skeletal muscle density and the presence of myosteatosis, were analyzed separately. Consequently, two models were created for multivariate analysis: model 1, which included age, BUN, AAS, and skeletal muscle density; and model 2, which included age, BUN, AAS, and presence of myosteatosis. In both models, elevated BUN (HR = 1.01, 95% CI: 1.00–1.02, *p* = 0.012 in model 1; HR = 1.01, 95% CI: 1.00–1.02, *p* = 0.019 in model 2) and high AAS (HR = 1.03, 95% CI: 1.01–1.04, *p* < 0.001 in model 1; HR = 1.02, 95% CI: 1.01–1.03, *p* = 0.002 in model 2) remained significant predictors. Additionally, skeletal muscle density in model 1 (HR = 0.91, 95% CI: 0.86–0.97, *p* = 0.003) and the presence of myosteatosis in model 2 (HR = 5.02, 95% CI: 1.09–23.04, *p* = 0.038) were significant contributors to poor OS.

**Table 4:** Univariate and multivariate Cox proportional hazard regression models for overall survival.

Variable	Univariate analysis			Multivariate analysis (Model 1)			Multivariate analysis (Model 2)		
	HR	95% CI	*p* value	adjusted HR	95% CI	*p* value	adjusted HR	95% CI	*p* value
Sex (male)	1.21	0.58, 2.54	0.613						
Age, year	1.04	1.00, 107	0.048	0.98	0.94, 1.02	0.378	1.00	0.96, 1.04	0.900
BMI, kg/m^2^	0.94	0.85, 1.03	0.185						
HTN (absence)	0.83	0.34, 2.05	0.690						
DM (absence)	1.79	0.79, 4.06	0.165						
VFA, cm^2^	1.00	0.99, 1.00	0.409						
SFA, cm^2^	1.00	0.99, 1.00	0.497						
SMI, cm^2^/m^2^	0.98	0.94, 1.02	0.307						
Visceral-to-subcutaneous fat ratio	0.96	0.74, 1.24	0.738						
Skeletal muscle area, cm^2^	0.99	0.98, 1.00	0.131						
Skeletal muscle density, HU	0.94	0.89, 0.98	0.009	0.91	0.86, 0.97	0.003			
Sarcopenia (absence)	1.25	0.59, 2.65	0.557						
Myosteatosis (absence)	5.66	1.34, 23.88	0.018				5.02	1.09, 23.04	0.038
AAS (100/m^2^ increment)	1.02	1.01, 1.03	< 0.001	1.03	1.01, 1.04	< 0.001	1.02	1.01, 1.03	0.002
Creatinine, mg/dL	1.00	0.91, 1.10	0.963						
eGFR, mL/min/1.73 m^2^	1.00	0.99, 1.02	0.846						
BUN, mg/dL	1.01	1.00, 1.02	0.001	1.01	1.00, 1.02	0.012	1.01	1.00, 1.02	0.019
Albumin, g/dL	0.57	0.29, 1.14	0.114						
CRP elevation (absence)	1.30	0.62, 2.75	0.489						

The reference category for each categorical variable is in the round brackets in the first column. Model 1, which included age, BUN, AAS, and skeletal muscle density; and Model 2, which included age, BUN, AAS, and presence of myosteatosis.

*BMI* body mass index, *VFA* visceral fat area, *SFA* subcutaneous fat area, *SMI* skeletal muscle index, *AAS* abdominal aortic calcium score, *eGFR* estimated glomerular filtration rate, *BUN* blood urea nitrogen, *CRP* C-reactive protein.

### Kaplan–Meier analysis for OS

**Figure 3:**
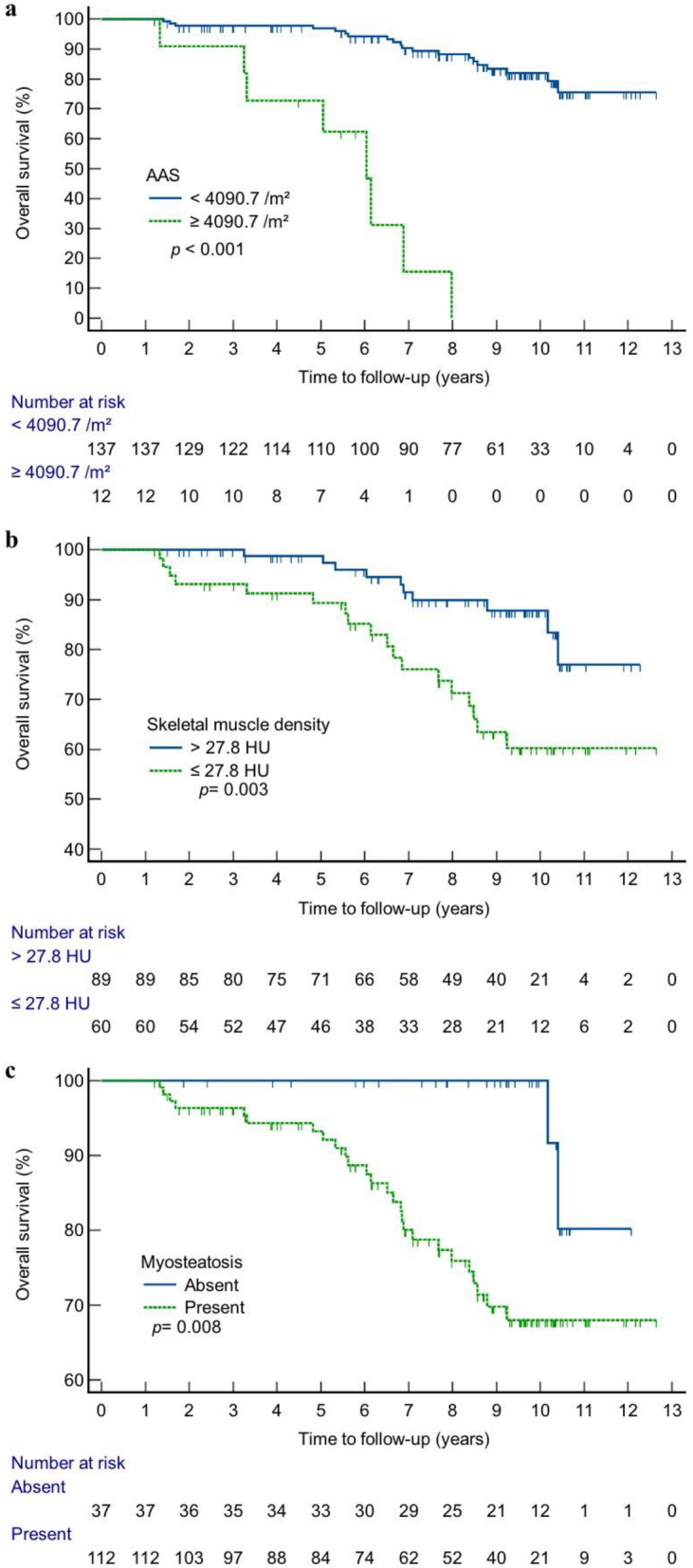
Kaplan–Meier curves according to abdominal aortic calcium score (AAS) and skeletal muscle density. (**a**) Kaplan–Meier curves grouped according to the calculated cut-off value of AAS. (**b**) Kaplan–Meier curves grouped according to the calculated cut-off value of skeletal muscle density. (**c**) Kaplan–Meier curves grouped according to the myosteatosis criteria.

Optimal cut-off values of the AAS and skeletal muscle density were 4090.7/m^2^ and 27.8 HU based on the maximized Youden’s index. In the Kaplan–Meier survival curves stratified by these optimal cut-offs, the patients with AAS > 4090.7/m^2^ and skeletal muscle density ≤ 27.8 HU experienced significantly shorter OS time (*p* < 0.001 and *p* = 0.003, respectively) (Fig. 3A, B). The group with myosteatosis also showed shorter OS time compared to the group without (*p* = 0.008) (Fig. 3C).

**Figure 4:**
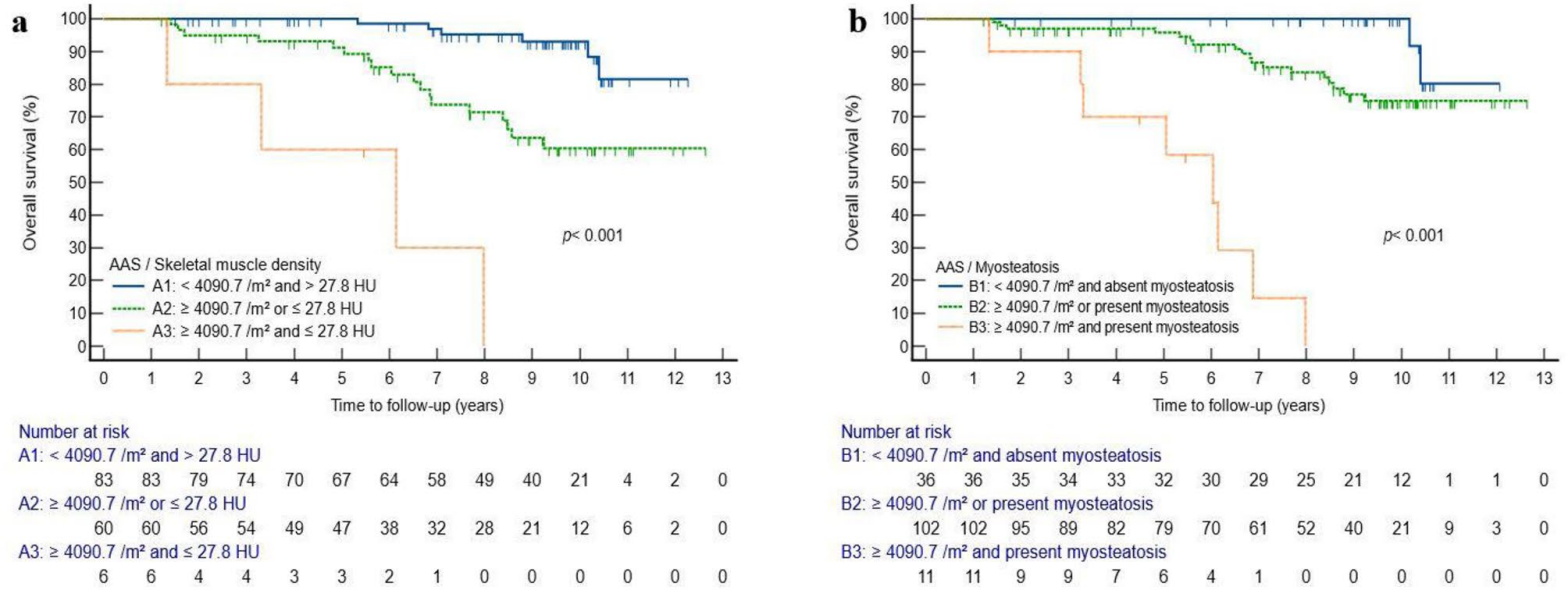
Kaplan–Meier curves categorized into three groups according to abdominal aortic calcium score (AAS) and skeletal muscle density. (**a**) Kaplan–Meier curves grouped according to the calculated cut-off value of AAS and skeletal muscle density. (**b**) Kaplan–Meier curves grouped according to the calculated cut-off value of AAS and the myosteatosis criteria.

When the cut-offs of the AAS and skeletal muscle density were combined, the patients were categorized into three groups: group A1 (AAS < 4090.7/m^2^ and skeletal muscle density > 27.8 HU, n = 83), group A2 (AAS ≥ 4090.7/m^2^ or skeletal muscle density ≤ 27.8 HU, n = 60), and group A3 (AAS ≥ 4090.7 /m^2^ and skeletal muscle density ≤ 27.8 HU, n = 6). The three groups showed significant different OS rates (*p* < 0.001) (Fig. 4A). Similar to the previous analysis, the patients were divided into three groups by combining the presence of myosteatosis and the cut-off value of AAS: group B1 (AAS < 4090.7 /m^2^ and absence of myosteatosis, n = 36), group B2 (AAS ≥ 4090.7 /m^2^ or presence of myosteatosis, n = 102), and group B3 (AAS ≥ 4090.7 /m^2^ and presence of myosteatosis, n = 11). Kaplan–Meier analysis showed significantly different OS rates among the three groups (*p* < 0.001) (Fig. 4B).

## Discussion

We showed the relationship between body composition data from abdominal CT and longitudinal changes in eGFR, as well as the relationship with OS in patients with non-dialysis CKD. The 149 patients with non-dialysis CKD had a high ratio of sarcopenia (53.0%) and myosteatosis (75.2%). High AAS, high baseline eGFR, and the presence of myosteatosis were significant prognostic factors for CKD progression. For OS, elevated BUN, high AAS, and either low skeletal muscle density or the presence of myosteatosis were independent factors for poor survival. Patients with CKD undergo non-enhanced abdominal CT scan for various reasons, and the advantage is that body composition on abdominal CT, specifically myosteatosis and abdominal aortic calcium score, can be use as biomarkers to predict the patient's prognosis without additional tests.

There have been several previous studies on body composition data and adverse clinical outcome in CKD patients^16–19^, but we are the first in our knowledge to review comprehensive data from multiple components of abdominal body composition, including fat, muscle, and vascular calcium scores in patients with CKD. These components interact mutually in various ways, emphasizing the importance of considering them simultaneously. For example, it has been suggested that higher fat mass may contribute to reduced muscle mass (i.e., sarcopenic obesity)^25,26^ and that sarcopenic obesity may be linked to chronic inflammation and increased vascular calcification^26,27^.

We discovered a relationship between myosteatosis and longitudinal renal function decline and OS in patients with non-dialysis CKD. Most previous studies investigating the association between muscle fat infiltration and adverse clinical outcomes in patients with CKD primarily focused on dialysis patients^8,9,28,29^. Although there have been reports that muscle fat deposition is related to muscle strength or function, to the best of our knowledge, our investigation is the first to examine myosteatosis and its negative clinical outcomes using abdominal CT scans in patients with non-dialysis CKD. One such study identified that increased echogenicity of the rectus femoris muscle on ultrasound was a predictor of muscle strength and physical performance in patients with non-dialysis CKD^30^. Another study examined muscle fat infiltration using mid-thigh MRI cross-sectional images and found a relationship between muscle fat infiltration and mitochondrial dysfunction in patients with non-dialysis CKD^31^.

The lack of association between the skeletal muscle area and adverse outcomes in our study is interesting and may reflect the importance of assessing skeletal muscle quality rather than just quantity. Recent studies suggested that myosteatosis plays an independent prognostic role from sarcopenia and may explain the controversial effects of skeletal muscle mass on various adverse clinical outcomes^22,32–35^. A research involving 1,974 community-dwelling adults from the Multi-Ethnic Study of Atherosclerosis examined the abdominal muscle area and density through abdominal CT scans and revealed that higher muscle density, not muscle area, was associated with a lower risk of all-cause mortality^35^. We also found a similar association with survival in patients with CKD, and we discovered a relationship between muscle density and renal function decline. The pathophysiological mechanism of myosteatosis in patients with CKD requires further investigation. However, current research suggests a multifactorial process, and it is believed that aging, poor nutritional status, inflammation, oxidative stress, and insulin resistance may be involved^28,36^.

Traditionally, the association between aortic calcification and CKD has been assessed using lateral lumbar X-rays^37^. Several studies showed an increase in the prevalence and severity of aortic calcification on lateral lumbar X-rays as CKD progressed in a non-dialysis population^38,39^. According to a recent study using abdominal CT, the AAS was shown to be associated with decreased eGFR in patients with non-dialysis CKD^16^. Our research is consistent with these findings and further demonstrates that the degree of aortic calcification itself is also associated with the long-term decline of renal function with a median follow-up period of 90 months. Our findings suggest that the AAS can be used to predict the prognosis of renal function in patients with CKD.

Although dual-energy X-ray absorptiometry (DXA) is widely used for body composition analysis, our results show the advantage of using abdominal CT for body composition analysis. DXA is limited in its ability to evaluate fatty infiltration in muscles and vascular calcification^40^. Considering the significance of myosteatosis and AAS, CT is likely to be additionally helpful as an imaging modality for body composition analysis in patients with CKD.

Study limitations should be mentioned. First, the retrospective nature of the research may introduce selection bias. Non-contrast abdominal CT scans were not performed on all CKD patients, and the reasons for these scans have differed among the patients; this could potentially impact the results. Second, our cohort only included a single ethnicity, making generalization to other ethnic groups difficult. The criteria used for defining sarcopenia and myosteatosis were based on non-Asian cohorts, and it might not be suitable for the Korean ethnicity. Third, the presence of protein in the urine indicates glomerular damages and the amount of protein excreted in the urine is known to be particularly important for predicting the CKD progression. However, we cannot include the urine protein into analysis due to retrospective nature of the research. Given that urine protein may serve as a possible confounder, further research is warranted. Fourth, the creatinine-based CKD-EPI equation was used for calculating eGFR, and it is possible that low muscle mass could lead to an overestimation of eGFR. Therefore, the lack of significant impact of skeletal muscle area and the presence of sarcopenia on CKD progression might be attributed to this factor. It is necessary for future research to use eGFR calculations using muscle mass-independent markers, such as cystatin C, to potentially overcome these limitations. Lastly, despite the increasing number of studies on machine learning-based CT body composition analysis, which is expected to play a significant role in utilizing opportunistic data in the future, our study employed a semiautomated method. Further research on risk stratification for patients with CKD using machine learning-based CT body composition analysis is necessary.

In conclusion, our study suggests the potential value of body composition data from abdominal CT scans in predicting renal function decline and OS in patients with non-dialysis CKD. The presence of myosteatosis and the high burden of aortic calcium were found to be prognostic factors for CKD progression and OS. Our findings may facilitate the utility of CT-based body composition data in clinical management of CKD patients.

## Data Availability

The datasets generated or analyzed during the study are available from the corresponding authors upon reasonable request.

## Abbreviations

CKD: Chronic kidney disease
ESRD: End-stage renal disease
CT: Computed tomography
VFA: Visceral fat area
SFA: Subcutaneous fat area
AAS: Abdominal aortic calcium score
eGFR: Estimated glomerular filtration rate
BUN: Blood urea nitrogen
HU: Hounsfield unit
SMI: Skeletal muscle index
IQR: Interquartile range
OS: Overall survival
OR: Odds ratio
HR: Hazard ratio
DXA: Dual-energy X-ray absorptiometry
